# Psychiatry's contribution to the public stereotype of schizophrenia: Historical considerations

**DOI:** 10.1111/jep.13011

**Published:** 2018-08-15

**Authors:** Heinz Katschnig

**Affiliations:** ^1^ Medical University of Vienna Vienna Austria; ^2^ IMEHPS, Research Institute for Social Psychiatry Vienna Austria

**Keywords:** dementia praecox, DSM, first rank symptoms, ICD, public stereotype, schizophrenia

## Abstract

The public stereotype of schizophrenia is characterized by craziness, a split personality, unpredictable and dangerous behaviour, and by the idea of a chronic brain disease. It is responsible for delays in help‐seeking, encourages social distance and discrimination, and furthers self‐stigmatization. This paper discusses the circumstances of the origins of the idea of a chronic brain disease (Emil Kraepelin, 1856‐1926), of the split personality concept derived from the term “schizophrenia” (Eugen Bleuler, 1857‐1939), and the craziness idea reflected in the “first rank symptoms”, which are all hallucinations and delusions (Kurt Schneider, 1887‐1967). It shows how Emil Kraepelin's scientific search for homogenous groups of patients with a common aetiology, symptom pattern, and prognosis materialized in the definition of “dementia praecox” as a progressing brain disease; how Eugen Bleuler's life and professional circumstances facilitated an “empathic” approach to his patients and prompted him to put in the foreground incoherence of cognitive and affective functioning, and not the course of the disease; finally, how Kurt Schneider in his didactic attempt to teach general practitioners how to reliably diagnose schizophrenia, neglected what Emil Kraepelin and Eugen Bleuler had emphasized decades earlier and devised his own criteria, consisting exclusively of hallucinations and delusions. In a strange conglomerate, the modern operational diagnostic criteria reflect all three approaches, by claiming to be Neo‐Kraepelinean in terms of defining a categorical disease entity with a suggestion of chronicity, by keeping Bleuler's ambiguous term schizophrenia, and by relying heavily on Kurt Schneider's hallucinations and delusions. While interrater reliability may have improved with operational diagnostic criteria, the definition of schizophrenia is still arbitrary and has no empirical validity—but induces stigma.

## 1 INTRODUCTION

Ever since I entered the Department of Psychiatry of the University of Vienna as a psychiatric trainee in 1968, I have struggled with understanding what is meant by the diagnosis of schizophrenia, and 50 years later I am not sure yet. I have tried, together with my colleagues, to describe and contrast all published definitions of the disorder,^1^ also introducing the concept of a “poly‐diagnostic” approach,^2^, ^3^ without coming to a conclusion. Over the decades, I have witnessed the demise of the traditional vague clinical concept of schizophrenia and its replacement by so‐called operational diagnostic criteria, ie, by providing checklists of symptoms and other criteria, first in 1980 by the American Psychiatric Association's Diagnostic and Statistical Manual III (DSM‐III),^4^ then by the World Health Organization with its Chapter V of the 10th revision of the International Classification of Diseases (ICD‐10) in 1992.^5^ Newer editions exist (DSM‐5^6^) or are being developed (ICD‐11^7^), but they do not deviate principally from their respective original operational versions.

All my professional life, I have witnessed the despair of patients and their family members when confronted with the diagnosis of schizophrenia. It is perceived as a virtual death sentence in terms of referring to a chronic, deteriorating, and incurable disease, to a “split personality” implying unpredictability, and to craziness symptoms, such as hallucinations and delusions, leading to dangerous behaviour. Since patients and family members know that everyone is thinking like this, the anticipated negative reaction by the social environment weighs heavily.^8^ There is a vast literature on the public stereotype,^9^, ^10^, ^11^, ^12^, ^13^ showing that it leads to social rejection and discrimination, causes delay in help‐seeking, and can give rise to self‐stigmatization.^14^ Being aware of the public stereotype, I observed myself and my colleagues, how we often tended to avoid as long as possible to communicate the diagnosis, knowing that all these elements were either completely wrong (split personality) or only potentially but not necessarily characteristic of the diagnosis of schizophrenia (chronicity; hallucinations and delusions). And I went in search of the roots of these elements in the history of psychiatry and am presenting here a chronicle of some better and some not so well‐known aspects, showing where they fit together and where they don't. A publication by Hoenig from 1983 was helpful for carrying out this search.^15^

The present paper is not on whether it is justified to conceive of schizophrenia as a disease entity and on the related logical and philosophical implications,^16^ and not on how (if at all) it should be defined. It is also not a paper on the sociological aspects of psychiatry as a profession, and the pressure on professions to build up a specific body of knowledge and skills owned exclusively by its members after a long training period (see the discussion of these aspects in Katschnig 2010^17^). A few remarks on the profession of psychiatry are nevertheless useful here for better understanding the whole story. That psychiatry had become a medical profession in the 19th century had not only to do with humanistic concerns but certainly also with the high professional status medicine enjoyed in society, not the least because of its scientific successes. The title of Jan Goldstein's treatise “Console and Classify—The French Psychiatric Profession in the Nineteenth Century”^18^ neatly captures this dual nature of the origins of psychiatry as a profession. Philippe Pinel, the legendary “liberator” of the mentally ill, was excited by the prospects a hospital provided for research, exclaiming in 1815^19^ *“What a source of instruction is provided by two hospitals of 100 to 150 patients each! …. What a varied spectacle of fevers or phlegmasias, malign or benign, sometimes highly developed in strong constitutions, sometimes in a slight, almost latent condition, together with all forms and modifications that age, mode of life, seasons, and more or less energetic morale affections can offer.”*

Not only did psychiatrists attempt to do as their medical colleagues did and define disease categories such as tuberculosis and scarlet fever, but it must also be remembered that all diagnostic systems developed in the first 150 years of psychiatry were based on assessing patients in mental hospitals, which was the dominant source of experience of the influential psychiatrists of that period. They saw a selection of patients at a stage of their disorder when they were most severely ill and disturbing for society, and this selection may have been different from hospital to hospital or country to country, which might explain some of the contradictory findings and suggestions of the leading psychiatrists of that period. Today, in times of community psychiatry the situation is entirely different, but elements of the old diagnostic concepts are still around.

Throughout the 19th century in many European countries, numerous attempts were made by professional “authorities” to define disease entities and design psychiatric classification systems. None of them found general acceptance in the profession, until, at the end of the century, the German psychiatrist Emil Kraepelin (1856‐1926), based on what he regarded as a scientific approach, bound together most of the loose ends into two major mental disorders, the chronic and progressing “dementia praecox” (to become “schizophrenia” soon afterwards), and the remitting “manic depressive insanity”. Until today, all professional discussions and controversies have focused on this dichotomy and the “firewall” erected by Kraepelin between them. The chronicity mark of “dementia praecox” became part of the public stereotype, although the concept of a progressing disease entity was challenged by many psychiatrists soon after its promulgation.

The central figure in this challenge was the Swiss psychiatrist Eugen Bleuler (1857‐1939), who laid the emphasis not on the early age of onset (Kraepelin's adjective “praecox”) and not on outcome (which he found to be varied in his patients), but on symptoms, and suggested in 1911 to change the term “dementia praecox” to “schizophrenia”. He thought that a neutral new term was useful for denoting the characteristic symptoms of the incoherence of association and affect which he regarded as essential for the diagnosis. Unfortunately, the term was misunderstood later outside psychiatry and became itself a part of the public stereotype.

The third significant contributor to the topic of the diagnosis of schizophrenia was the German psychiatrist Kurt Schneider (1887‐1967), who in 1939, several decades after the seminal publications of Kraepelin and Bleuler, defined specific hallucinations and delusions as the “first rank symptoms” of schizophrenia. He was cautious and modest and would not agree that schizophrenia could be conceived as a disease entity in the medical sense but rather as a “psychopathological type”, and he did not do much to spread his ideas. However, 40 years later these “craziness symptoms” were enthusiastically picked up in the second half of the 20th century by professional psychiatry and became centre stage in the definition of schizophrenia in today's major operational diagnostic systems, first in the DSM‐III in 1980, then in the ICD‐10 in 1992.

## 2 KRAEPELIN (1856 – 1926): “DEMENTIA PRAECOX” AND CHRONICITY

It was a rather strange move. In 1886, at the age of 30, an aspiring young psychiatrist from Germany was appointed to the chair of psychiatry at the University of Dorpat (then in Russia; today Tartu in Estonia), a place where the vast majority of the population spoke languages he did not understand and also could not acquire quickly—namely Estonian, Russian, and Latvian. When I read his memoirs, I was particularly struck by the following passage^20^: *“The majority of the ordinary patients spoke and understood only Estonian .... I was, therefore, unable to communicate with most of them without continuous interpreting ….. it was impossible for me to perceive slight deviations in pronunciation, ways of expressing a thought or formation of words or sentences.”*

Kraepelin worked in Dorpat from 1886 to 1891. It cannot be excluded that the communication restraints in Dorpat have stimulated Kraepelin's interest in the history and the course of the illness, which he began there to pay attention to and which led him, as he also writes in his memoirs, to the postulation of a degenerative process occurring in what he later called “Dementia praecox”.^21^ After leaving Dorpat in 1891 to take up the psychiatric chair at the University of Heidelberg, Kraepelin started a “Research Programme” whose objective was the creation of a stable description and classification of the psychoses, ie, the identification of homogeneous groups of patients who had the same aetiology, course, duration, and outcome.^22^ The scientific tool by which he attempted to reach this objective was an elaborate documentation system with “index cards”, containing condensed information on each patient, which he grouped under different aspects, calling the results of this approach later on *“the victory of scientific observation over philosophical and moral meditation”*.^23^ Two years after arriving in Heidelberg, Kraepelin published the 4th edition of his textbook,^24^ where he introduced the concept of “Dementia praecox” as an irreversible, deteriorating, and incurable disease starting early in life and subsumed it under the heading of “Degenerating psychological processes”.^21^

In the 6th edition of his textbook, published in 1899,^25^ Kraepelin finally contrasted “Dementia Praecox” with “Manic‐Depressive Insanity” (Manisch‐Depressives Irresein), based mainly on the observations of a different course of the latter, ie, an episodic course with remission in between the episodes, while “dementia praecox”, according to Kraepelin, was starting early in life and progressing, although at different speeds. By this dichotomy, he put a “firewall” between the two which is still being debated nowadays. As far as “dementia praecox” is concerned, Kraepelin concluded that *“The vast majority of mentally handicapped and semi‐handicapped after dementia praecox gradually accumulate in the big mental asylums (Heil‐ und Pflegeanstalten); indeed, these patients, because they do not die off quickly and often spend their whole lives in the asylum, constitute the bulk of the insane requiring care“*.^26^ At a later stage in his life, Kraepelin conceded that there were also a few cases of complete remission in dementia praecox, but this did not lead him to revise the diagnosis.^15^

Kurt Schneider, whose decisive influence on the definition of schizophrenia will be discussed below, said much later that Kraepelin's concept of the human person was that of the positivist natural sciences of the 19th century where he looked only from outside on the psychotic person.^27^ This assessment corresponds to that of a later scholar of Kreapelin, and his work who pointed out that Kraepelin showed a lack of empathy with his patients (while he cared humanely for their physical well‐being).^28^

For the subject of the present paper, it is important to note that the “chronicity” aspect is the one issue for which Kraepelin is best remembered. Public beliefs still give a poor prognosis to schizophrenia,^13^ in any case, a poorer one than to depression.^29^ Today, it is obvious—and Eugen Bleuler^30^, ^31^ pointed this out already a few years after Kraepelin's seminal publication—that Kraepelin described only one “end” of the spectrum of schizophrenia, the group of patients, whose disorder did not improve and even showed a progressive course. Today, the common finding of large long‐term studies is that only about one third of first episode patients may belong to the “chronic group” and that recovery is not infrequent.^32^, ^33^, ^34^, ^35^ It is difficult to come to a definite conclusion though, since the contexts have changed completely over time, with most patients living outside institutions (“hospitalism” and “institutionalism” may have played a big role 100 years ago^36^), the empirical outcome criteria studied being wide‐ranging (covering not only symptoms but also functioning and disabilities), and new treatments having become available.

## 3 EUGEN BLEULER (1857‐1939): THE TERM “SCHIZOPHRENIA” AND ITS MISINTERPRETATION AS “SPLIT PERSONALITY”

Kraepelin's concept of Dementia praecox was soon criticized, first for its chronicity criterion, which was suggested to be overrated, and, second, for neglecting psychopathological theory and presenting only an “unstructured mosaic of symptoms”.^37^ What was neglected by Kraepelin became a major concern of Eugen Bleuler, a Swiss psychiatrist, just one year younger than Kraepelin, who had worked in several psychiatric hospitals in Switzerland, before he became director of the “Burghölzli”, the psychiatric university hospital of the city of Zürich, in 1898, where he stayed until his retirement in 1927.

Bleuler introduced the term schizophrenia first in 1908.^30^ In 1911, the lengthy monograph “Dementia praecox oder Gruppe der Schizophrenien” (“Dementia praecox or the group of schizophrenias”)^31^ was published. The title was most carefully devised. It politely uses Kraepelin's term “Dementia praecox”, linking it to Bleuler's two suggested alternative concepts with the word “or”. The alternative concepts are represented, first, by the word “group” and the plural used, suggesting that the disorder has several forms of manifestation (especially concerning the age of onset and the course), and second, by the term “schizophrenias”, signalizing Bleuler's focus on psychopathology, actually “rejecting” Kraepelin's concept of “dementia praecox” and making a complete conceptual U‐turn.

Eugen Bleuler had a different mindset than Kraepelin. He regarded himself not so much as a scientist, who attempts to design a psychiatric classification system but as a doctor who talks with his patients and wants to help them. His statement *“Someone who becomes a doctor … will be able to help the individual patient better than a doctor who is not able to talk with a patient and is more interested in science than in the individual patient”* is reported by Daniel Hell,^38^ a successor of Eugen Bleuler at the Burghölzli, who provides an account of some additional aspects of Bleuler's private and professional life, which can shed some light on his specific mindset.

First, it is interestingly stressed that Eugen Bleuler married rather late (in 1899 at the age of 43, soon after he had become director of the “Burghölzli” a year earlier) and is said to have had therefore much time in his early professional years to spend with his patients at the Swiss Hospital Rheinau (where he was director for 12 years from 1986 to 1898)—*“he lived with his patients, 14 to 16 hours a day and work on Sundays were not rare”*. Second, he had an elder sister with a mental illness with whom he had lived for some time at home in the family (in fact, not far from the Burghölzli hospital), and whom Bleuler took to live with him and his family in the director's apartment. Third, he was able to speak with his patients in the local Swiss language, which is a very peculiar German dialect, not easily grasped by foreigners (his predecessors in the Burghölzli, some of whom were Germans, must have had similar problems of understanding their patients as Kraepelin had in Dorpat). Finally, for some time, he showed interest in psychodynamic issues, with Carl Gustav Jung being one of his assistants and with correspondence with Sigmund Freud.

Taking all these aspects together, it is somewhat understandable that Bleuler has been regarded as an empathic person and that his focus was directed to the more subtle aspects of the psychological functioning of his patients. Put simply, the central pathological feature he described for schizophrenia was the incoherence of psychic functions in the cognitive and affective areas (in the absence of obvious organic brain dysfunction), manifested among others by the loosening of associations, and the term schizophrenia was shorthand for these subtle psychopathological abnormalities. A few other psychiatrists had already made similar observations and had proposed other terms “for replacing dementia praecox” (eg, “dysphrenia”^39^ and “intrapsychic ataxia”^40^). Bleuler discusses these terms and concludes that no suggestion is better than his own, namely the completely new word schizophrenia, since there would be no danger of misunderstandings. Unfortunately, that is exactly what happened later.

What became equally important as Bleuler's focus on the incoherence of association and affect, which he called “basic symptoms”, was that Bleuler relegated hallucinations and delusions to a subordinate status by classifying them as “accessory symptoms”, which he regarded as not relevant for the diagnosis since they could be present or absent.

Other than the word “schizophrenia”, Bleuler‘s basic symptoms did not catch on permanently in psychiatric classification systems. They are obviously too difficult to assess reliably, and the descriptions of symptoms are often mixed with interpretations which leads to a lack of clarity.^41^ They still played some role in the DSM‐I (1952) and DSM‐II (1968), as well as the ICD‐8 (1967) and ICD‐9 (1978), but were downgraded in the operational versions, ie, DSM‐III (1980) and ICD‐10 (1992), where hallucinations and delusions gained top status for diagnosing schizophrenia, in complete contradiction to Bleuler's concept where they were only “accessory”.

What remained from Bleuler, both for the diagnostic systems and for the public stereotype, is the word schizophrenia. By using the old Greek word “phren” for the mind and “schizein” (= to fall into pieces, not only split), Bleuler wanted to express the disintegration and incoherence of psychological functions (thinking, affect) and not the separation into two entities. A couple of decades later, the term became publicly used by the media in the sense of “conflicting nature” and “contradictoriness”, and also as a “split personality”.^42^ And this heavily affects patients in the public stereotype since it is related to unpredictability and dangerousness.^43^ It seems though that this component of the stereotype is restricted to the educated segment of the general public.^44^ The latter finding is important in practical terms—regulations and laws, which may contain the discrimination of mentally ill persons, are formulated and applied by persons with better education. In a survey in Austria,^12^ medical doctors had the highest rate of misunderstanding the term schizophrenia—given the authority of physicians, one can imagine how detrimental this can be for patients and their families.

Psychiatrists are still struggling with communicating the diagnosis to patients and family members and in some places the term has been officially abandoned, such as in Japan, where the original translation of schizophrenia “Seishin Bunretsu Byo” (“mind‐split‐disease”) was replaced by “Togo Shitcho Sho” (“integration disorder”; which is in fact what Eugen Bleuler wanted to express with the term “schizophrenia”). It was subsequently shown that Japanese psychiatrists increased the rate of informing the patient about the diagnosis from 37% to 70% within three years after the change.^45^, ^46^ Opinions are divided though on the issue of renaming the disorder.^47^

## 4 KURT SCHNEIDER (1887‐1967): “FIRST RANK SYMPTOMS” AND CRAZINESS

It took a few decades until the next U‐turn. Delusions and hallucinations, downgraded by Eugen Bleuler to “accessory symptoms” in 1911, were upgraded to the top by the German psychiatrist Kurt Schneider in a brochure for general practitioners (!) published in 1939.^48^ There, he suggested seven types of hallucinations and delusions which he called “First Rank Symptoms”. They included audible thoughts, voices arguing and/or discussing, voices commenting, somatic passivity experience, thought withdrawal/influenced thought, thought broadcasting, and delusional perceptions (“made volition” was added as the eighth symptom in a later publication). And Kurt Schneider added: *“If they are present without a doubt and no underlying physical disease can be identified, then we clinically speak in all modesty of schizophrenia”*.

At the time, the publication remained practically unnoticed because of the Second World War. But also after the war, when Kurt Schneider came to be the chair of the University of Heidelberg, the impact of his suggestion to use specific delusions and hallucinations for diagnosing schizophrenia was very limited. However, after a long latency period, the situation changed in the 1960s and 1970s due to several developments in psychiatry, which will be discussed below—the “First Rank Symptoms” were suddenly highly appreciated by American and international psychiatry and found a prominent place in the classification systems 40 years after their first publication.

Kurt Schneider (1887‐1967), a contemporary and admirer of the philosopher Karl Jaspers (1883‐1969)—who set a landmark for phenomenological descriptions of abnormal psychological phenomena with his “General Psychopathology”^49^—was director of the Clinical Unit of the German Research Institute for Psychiatry (Deutsche Forschungsanstalt für Psychiatrie) in Munich since the early 1930s. He was a clinician and not a researcher, published rather little, but had a definite didactic interest.

After using the term “First Rank Symptoms” for the first time at a conference in Berlin in 1938, he wrote the above‐mentioned brochure for general practitioners in 1939. In the introduction, he says: *“To publish a short and scientifically not particularly highbrowed article as a monograph, requires justification: My justification is this: I hope that I can achieve my intention better than by getting it out in a journal. I intend to help the general practitioner in making a psychiatric diagnosis. These guidelines aim at those psychiatric diagnoses, where the general practitioner depends completely on psychopathological symptoms, ie, schizophrenia and cyclothymia. It is my experience that general practitioners very frequently overlook exactly those diagnoses. I want to point out at least the most frequent errors occurring when psychopathological phenomena are to be elicited and used for making a psychiatric diagnosis. At the end of this monograph, I attempt to establish a rank order of psychopathological symptoms, which might also be of interest to psychiatrists.”*.^48^

Given the later worldwide relevance of the “First Rank Symptoms” the formulation at the end *“which might also be of interest to psychiatrists”* is especially remarkable. Kurt Schneider was an unassuming man. In the year 1950, when his book “Clinical psychopathology” (containing the “First Rank Symptoms”) was published in its third edition,^50^ he wrote to a colleague: *“In reality, I do not believe anymore in the correctness of what I am teaching. I have probably reached now what Jaspers calls the inevitable final point: failure”*.^51^ And a few years later in a similarly minded letter to Karl Jaspers: *“Psychopathology plays only a modest role nowadays, in some hospitals no role at all”*.^52^

While by raising delusions and hallucinations to the top and not mentioning “basic symptoms” at all Kurt Schneider dissented from Eugen Bleuler, but by not setting any criterion for the duration of schizophrenia, he followed him and disagreed with Emil Kraepelin. Also, he did not believe in psychiatric disease entities as Kraepelin had done and suggested to “free psychiatry from the slavery of neurology”—all in stark contrast to the inclusion of the “First Rank Symptoms” in the “Neo‐Kraepelinean” DSM‐III definition of schizophrenia which will be discussed in more detail below.

Kurt Schneider regarded himself as a phenomenologist and psychopathologist focusing on the inner psychological experiences of patients in order to find “types of human reactions”, and strove for finding “practical types”. General practitioners without specific psychiatric training and clinical experience could detect schizophrenia if a patient simply reported the specific hallucinations and delusions. In a letter to Jaspers, criticizing somewhat the philosopher, he had written already in 1923: *“Methodological subtleties are not enough, one also has to show that it is useful for something”* (all quotes above are from^52^). And also: *“This evaluation is only relating to the diagnosis. It does not say anything concerning the theory of schizophrenia as did Bleuler's ‘basic symptoms’ and ‘accessory’ symptoms … One might also acknowledge other first rank schizophrenic symptoms. But we confine ourselves to those, which can be identified without too much difficulty”* 53, p., 129). Reliability over validity! Something must ring in the ears of those familiar with the development of the operational diagnostic criteria in the 1980s and afterwards.

## 5 DSM‐III AND ICD‐10: WHY HAVE HALLUCINATIONS AND DELUSIONS BECOME SO PROMINENT?

Kurt Scheider died in 1968 at the age of 80. In an obituary, it was emphasized that *“he was not interested in the short‐ or long‐lived clinical entities which are so popular with psychiatrists”*.^54^ But his “First Rank Symptoms” were exactly used for defining such clinical entities. After a long latency period, delusions and hallucinations received high visibility and considerable attention by their inclusion as the leading symptoms for diagnosing schizophrenia in the operational diagnostic systems. Hallucinations and delusions are equivalent to craziness, representing a complete detachment from “reality” and have become a core component of the public stereotype of schizophrenia.

The introduction of DSM‐III in 1980 was dubbed the “Neo‐Kraepelinean revolution” in American psychiatry^55^ in the sense that it described discrete psychiatric disease entities and brought psychiatric classification and diagnosis back into medicine, after the preceding editions DSM‐I (1952) and DSM‐II (1968) had been strongly influenced by psychodynamic thinking.^56^ In the operational diagnostic formulation of schizophrenia, the “First Rank Symptoms” of Kurt Schneider figured most prominently among the psychopathological criteria. From Eugen Bleuler's concepts, nothing but the misleading term schizophrenia remained. So, they are all here: Kraepelin, Bleuler, and Schneider. But how did the “First Rank Symptoms” make it to the top, 40 years after their original publication?

In the 1950s, two significant developments occurred which would change psychiatry forever and which became of eminent relevance for the diagnostic formulation of schizophrenia in the operational diagnostic systems in use until today. First, the so‐called neuroleptics (today more appropriately called “antipsychotics”) were discovered,^57^ second, the need for standardized assessment of psychopathological phenomena became apparent in psychiatry. The “First Rank Symptoms” of Kurt Schneider, dormant since 1939, fit perfectly well to these developments, once they had become known in the English‐speaking world by the translation of his book “Clinical Psychopathology” in 1959.^58^

The neuroleptics (later called antipsychotics) worked mainly against hallucinations and delusions and not so well against other symptoms of schizophrenia, such as withdrawal and inactivity; they could even worsen them. One suggestion is that this finding might have over time nudged psychiatrists towards moving hallucinations and delusions up in diagnostic algorithms. Psychopharmacologists became fond of psychotic symptoms and doubted Eugen Bleuler's approach of ranking hallucinations as accessory symptoms. For instance, Paul Janssen, the well‐known founder of a pharmaceutical company, which later on produced and successfully marketed antipsychotics (such as haloperidol and risperidone) said about Bleuler's schizophrenia concept: *“The aetiology of schizophrenia is Dr Bleuler”* 22, 59 . It is perhaps not too farfetched to assume that the antipsychotics played a significant role when later on committees decided to put Kurt Schneider's psychotic symptoms on the top of the criteria for diagnosing schizophrenia in the DSM‐III. In this connection, it is noteworthy that the British psychiatrist and psychopathologist Frank Fish, who was well known in the 1960s, had a thorough knowledge of the German clinical literature of the first half of the 19^th^ century, and (in addition to Kurt Schneider's work) also had known the so‐called Leonhard classification of schizophrenia,^60^ made the observation that the new neuroleptic drugs worked better in one subgroup (the so‐called non‐systematic schizophrenias) than in the systematic schizophrenias.^61^ Fish died early and these and other interesting observations did not play a role for the diagnostic formulation of schizophrenia in the new classification systems. Kurt Schneider's easy to grasp first rank symptoms became dominant.

The second change in psychiatry was that standardization and quantification in the assessment of psychopathological phenomena became a need in the 1950s and later. The increasing number of psychopharmacological studies required standardized quantifiable measures of psychopathology, and rating scales started to spring up.^62^, ^63^ But also a general need for standardized assessment of psychopathological phenomena was increasingly voiced, since *“a more serious obstacle of progress in psychiatry is difficulty of communication”*, as Stengel said in a paper published in the Bulletin of the World Health Organization in 1959.^64^ Understandably, the World Health Organization was especially sensitive to such difficulties in communication across countries. Also, clinical and epidemiological psychiatrists started to note the need for standardization, showing, for instance, how psychiatric diagnoses differed between New York and London.^65^ From the 1960s onwards, several attempts started to standardize the assessment of psychiatric patients' symptoms and diagnoses by developing structured assessment schedules and interviews with definitions of terms and scoring systems,^66^, ^67^ later on also including rules how to derive psychiatric diagnoses by using computer algorithms.^68^

Schneider's “First Rank Symptoms” became more and more prominent in research on schizophrenia.^69^, ^70^ The straightforwardness of assessing their presence or absence was probably an important reason for their increasing attractiveness—if the patient was reporting them, then schizophrenia was present, and everyone would agree. Gone were Bleuler's basic symptoms—they were very difficult to elicit anyhow and needed a close understanding of the language and the way a patient was thinking, and reliability would be very low. The “Research Diagnostic Criteria (RDC)”,^71^ the first elaborate system of criteria for psychiatric diagnoses for research (preceded by and based on the less elaborate “St. Louis criteria”^72^) practically imitated Kurt Schneider's approach of listing symptoms (two of eight symptoms were required, whereby seven of the eight were hallucinations and delusions closely related to Schneider's “First Rank Symptoms”). Quantification and measurement had arrived in psychiatry and had not spared schizophrenia. High interrater reliability became the new god, and validity, in the sense that the defined disorders represented “natural kinds” of diseases, was not a topic anymore.

When in 1980, the First Rank Symptoms were incorporated prominently into the DSM‐III schizophrenia definition (now one of six symptoms was enough for making the diagnosis, and of the six, five were hallucinations and delusions), no one seemed to have cared anymore about a theory of schizophrenia. Seymour Kety, an eminent researcher on the biology of schizophrenia, brought this to the point by saying^73^:*”Features regarded by both Kraepelin and Bleuler as fundamental and characteristic (impoverishment of affect, disturbances in personal contact and rapport, ambivalence, lack of motivation, depersonalisation, and stereotypes) were specifically rejected, and the new criteria were restricted to particular types of hallucinations and delusions which Bleuler had regarded as accessory symptoms. Schneider established a new syndrome with features that are more easily perceived and described, and which therefore show a higher degree of inter‐rater reliability, features which are economically put into checklists and fed into computers. That syndrome may be more prevalent, have a more favourable outcome, and be more responsive to a wide variety of treatments, but it is not schizophrenia.”*

## 6 CONCLUSION

The diagnosis of schizophrenia has become an atheoretical conglomerate in the large classification systems of the DSM and ICD, combining definitional elements from historical descriptions which had not been thought of as belonging together by their authors. On the contrary: As described above, Eugen Bleuler made a U‐turn after Kraepelin, and Kurt Schneider criticized Kraepelin as a 19^th^ century positivist natural scientist^27^ and upgraded Bleuler's accessory symptoms to “first rank” symptoms.

Apart from several other types of criticism of the DSM approach,^22^, ^74^ it has to be noted from an anti‐stigma perspective that, without primarily intended by Kurt Schneider in 1939 (“later they might become of some interest to psychiatrists”), “craziness symptoms” are prominent today in the symptom pattern of schizophrenia in the operational diagnostic systems (although somewhat less so in more recent editions, where other symptoms, such as inactivity and thought disorders, have slightly moved up in the hierarchy of symptoms); that from Eugen Bleuler the stigmatizing term “schizophrenia” remained, with its public perception as “split personality”; and that the concept of Kraepelin's disease entities characterizes the whole DSM with the schizophrenia criterion of a “minimum duration of symptoms for 6 months” hinting at chronicity.

Year by year worldwide, scores of people receive the diagnosis of schizophrenia and attempt to find out what is meant by the label, but also hide it in order to avoid the negative consequences for their lives because of the public stereotype resulting from a scientifically unjustified disease conglomerate. This is not to say that there are no people who at some stages of their life experience one or the other or several psychopathological phenomena or criteria contained in the operational definition of schizophrenia, sometimes for a short, sometimes for a prolonged period. It is the categorical disease entity “schizophrenia” such as it has been constructed from psychopathological phenomena and the consequences of this label for people's lives, which is the problem.

Millions of copies of the several DSM editions have been sold to the general public, and today internet searches provide information about these criteria easily to everyone. It is true that some websites warn of the “myths about schizophrenia”,^75^ but others reinforce it (eg, “Schizophrenia DSM‐5 Definition—Schizophrenia is a severe and chronic mental disorder, characterized by disturbances in thought, perception and behaviour”^76^). Today, a multitude of efforts are underway to combat the stereotype, by fighting the chronicity prejudice with the recovery concept,^77^ the split personality idea with education and renaming the disorder,^45^, ^78^, ^79^ and the implications of hallucinations and delusions with behavioural interventions.^80^, ^81^ It seems though that the categorical disease concepts of mental disorders are embedded in a hermetic professional system and the term schizophrenia, like other diagnostic labels, will continue to be used without questioning it—in textbooks for educating medical students, in clinical guidelines for practitioners, in research (although it has been shown that the diagnostic algorithms produce heterogeneous groups of patients), in hospital payment systems (although the diagnosis explains only a fraction of the cost variance), and in health statistics. Alternative approaches to using the categorical disease entity “schizophrenia”, such as the multidimensional and the person‐centred approaches, or the use of a vulnerability stress coping model, can be considered and may in fact be practised by psychiatrists here and there. The question is whether they can be systematically adopted by the profession of psychiatry given its medical self‐definition as well as the fact that, for several other reasons, psychiatry as a profession might face an uncertain future.^17^
